# Convolutional-neural-network-based diagnosis of appendicitis via CT scans in patients with acute abdominal pain presenting in the emergency department

**DOI:** 10.1038/s41598-020-66674-7

**Published:** 2020-06-12

**Authors:** Jin Joo Park, Kyung Ah Kim, Yoonho Nam, Moon Hyung Choi, Sun Young Choi, Jeongbae Rhie

**Affiliations:** 1 Department of Radiology, St. Vincent’s Hospital, College of Medicine, The Catholic University of Korea, Seoul, Republic of Korea; 2 Division of Biomedical Engineering, Hankuk University of Foreign Studies, Gyeonggi‐do, Republic of Korea; 3 Department of Radiology, Eunpyeong St. Mary’s Hospital, College of Medicine, The Catholic University of Korea, Seoul, Republic of Korea; 4 Department of Radiology and Medical Research Institute, School of Medicine, Ewha Womans University, Seoul, Republic of Korea; 5 Department of Occupational and Environmental Medicine, College of Medicine, Dankook University, Cheonan, Republic of Korea

**Keywords:** Gastrointestinal diseases, Diagnosis

## Abstract

Acute appendicitis is one of the most common causes of abdominal emergencies. We investigated the feasibility of a neural-network-based diagnosis algorithm of appendicitis by using computed tomography (CT) for patients with acute abdominal pain visiting the emergency room (ER). A neural-network-based diagnostic algorithm of appendicitis was developed and validated using CT data from three institutions who visited the ER with abdominal pain and underwent abdominopelvic CT. For input data, 3D isotropic cubes including the appendix were manually extracted and labeled as appendicitis or a normal appendix. A 3D convolutional neural network (CNN) was trained to binary classification on the input. For model development and testing, 8-fold cross validation was conducted for internal validation and an ensemble model was used for external validation. Diagnostic performance was excellent in both the internal and external validation with an accuracy larger than 90%. The CNN-based diagnosis algorithm may be feasible in diagnosing acute appendicitis using the CT data of patients visiting the ER with acute abdominal pain.

## Introduction

Acute appendicitis is one of the most common causes of abdominal emergencies involving abdominal pain^1–3^. The surgical procedure is still a representative treatment, although nonoperative management with antibiotics has been considered as an alternative treatment for uncomplicated appendicitis^4^. The diagnosis of acute appendicitis is still challenging, although many studies have been performed. Misdiagnosis or delayed diagnosis increases the incidence of perforation, peritonitis and negative laparotomy, which are associated with morbidity and mortality^1^. Therefore, a quick and accurate diagnosis of acute appendicitis is necessary for efficient clinical care of acute abdominal pain. However, diagnostic errors are common because symptoms are frequently unspecified and overlap with other diseases. The diagnosis of acute appendicitis is difficult even after physical examination by an expert and with laboratory findings^1^. To improve the diagnostic performance, clinical scoring systems such as the Alvarado score, pediatric appendicitis score, appendicitis inflammatory response score and RIPASA score have been proposed to stratify patients with suspected appendicitis based on specific symptoms, signs, and laboratory data^5–7^. These score systems are helpful in the decision process, but their use as an independent diagnostic tool is controversial^8^. Therefore, imaging modalities such as ultrasound (US) and computed tomography (CT) have played an important role in diagnosing acute appendicitis^9^. US is a widely used diagnostic technique. However, US studies might be limited, with various diagnostic accuracies due to many causes, such as poor operator skill, abundant bowel gas, obesity, and anatomic variation, and limitations in exploring patients with previous laparotomies^10^. CT is considered the gold standard for evaluating acute appendicitis^3^. It is an objective study technique, compared with US, that is operator dependent, but experts trained on radiologic imaging are necessary for proper interpretation.

Currently, deep learning methods have been developed and validated for medical image classifications that allow a machine to receive image data as input and to automatically discover the image representations needed for detection or classification^11^. If a deep-learning-based algorithm is capable of interpreting CT images at the radiologist level, it can compensate for the absence of the radiologist without delayed diagnosis or misinterpretation, especially in an emergency.

In this study, we investigate the feasibility of a convolutional neural network (CNN)-based diagnosis algorithm of acute appendicitis using abdominopelvic CT for patients with acute abdominal pain who visited the emergency room (ER).

## Methods

This study was performed in accordance with the Declaration of Helsinki. The institutional review boards of the three institutions (St. Vincent’s Hospital, Eunpyeong St. Mary’s Hospital, Ewha Womans University Medical Center) considered here approved this study. Informed consent was waived due to the retrospective nature of this study.

### Patients and dataset

For training and internal validation, a CT dataset with acute appendicitis findings was collected from patients who visited the emergency department with acute abdominal pain between December 2018 and May 2019, underwent abdominopelvic CT during the medical care process in the ER and were diagnosed with acute appendicitis clinically, which was then confirmed as acute appendicitis pathologically through surgery. CT image sets showing a normal appendix were included for approximately twice the number of appendicitis cases among patients who visited the emergency department with acute abdominal pain during the same period and underwent abdominopelvic CT, though with no abnormalities found in the appendix. Patients who underwent surgical removal of the appendix and had a tumor in the appendix were excluded. Cases involving CT examination with image degradation beyond a moderate level due to artifacts introduced by motion or metal materials and cases with a urinary stone as the cause of abdominal pain were excluded. All CT images for training were obtained using a 64-slice CT scanner (Discovery CT 750 High Definition, GE Healthcare). CT examinations were performed in helical mode. A tube voltage of 100 kVp and an automatic tube current modulation technique were used. The section thickness was 3.75 mm, and the section interval was 3.75 mm.

For external validation of a trained CNN-based algorithm, CT image sets obtained under the same clinical setting from two institutions between April 2019 and June 2019 were selected. CT images for external validation were acquired using two different CT scanners (Somatom Definition Edge, Siemens for institution 1; Somatom Perspective, Siemens for institution 2). For institution 1, CT examinations were performed in helical mode. A tube voltage of 100 kVp and an automatic tube current modulation technique were used. The section thickness was 5 mm, and the section interval was 5 mm. For institution 2, CT examinations were performed in helical mode. A tube voltage of 110 kVp and an automatic tube current modulation technique were used. The section thickness was 3 mm, and the section interval was 3 mm.

### CNN-based algorithm

**Figure 1:**
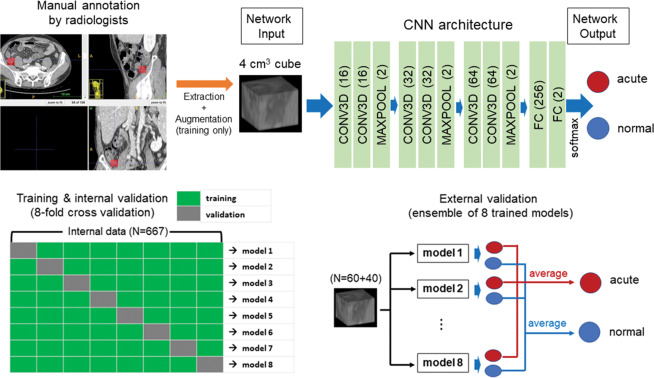
Schematic overview of a deep convolutional neural network approach for the diagnosis of acute appendicitis (upper) and the validation process (lower). For internal validation, 8-fold cross validation was used. The dataset was randomly separated into 8 datasets. Hyperparameters such as the learning rate, the number of epochs, and the number of layers were determined during the first model training and then used to train the other 7 models. The remaining 1/8 of the data were used for a test. For external validation, a CT dataset acquired from two institutions was used for the 8 differently trained CNN models. The final classification of acute appendicitis through a CNN was determined using the average of the network outputs [drawn by ITK-SNAP (version 3.6; http://www.itksnap.org/pmwiki/pmwiki.php) and Microsoft PowerPoint].

For all CT image sets, 3D isotropic cubes (4 × 4 × 4 cm^3^) including the appendix region were manually annotated and extracted using an open-source free software (ITK-SNAP, version 3.6; http://www.itksnap.org/pmwiki/pmwiki.php)^12^. For internal validation, manual extraction was performed by an abdominal radiologist with 12 years of experience. Each image set was labeled as acute appendicitis or a normal appendix. The deep CNN used in the algorithm was built with six convolutional layers, three max-pooling layers and two fully connected layers, as described in Fig. 1 (upper). After the two consecutive 3D convolutional layers (kernel size was 3 × 3 × 3), the rectified linear unit and 3D max-pooling (kernel size was 2 × 2 × 2) operation were applied to the output of convolution. The number of channels in all the convolutional layers was 16, 16, 32, 32, 64, and 64, in that order. The numbers of nodes for the fully connected layers were 256, and 2. A 3D CNN was trained via a supervised localization approach as an annotated portion to perform binary classification on the input 3D images. In the training process, a cross-entropy function was used as the loss function of the network, and the kernel size was 3 for all convolutional layers. To reduce overfitting, several data augmentation processes, such as shifting, flipping, and adding random noises, were applied. A fully connected layer generated the output. The softmax function was applied to the output value, and two numerical values, of which the sum was 1, were calculated as the image-level probability of acute appendicitis. For assessment of the CNN algorithm performance, 8-fold cross validation was conducted. The entire dataset used for internal validation was randomly separated into 8 datasets. Hyperparameters such as the learning rate, the number of epochs, and the number of layers were determined during the first model training, and the same parameters were used to train the other 7 models. For each model, 7/8 of the data were used to update the network parameters with the same hyperparameters, and the remaining 1/8 of the data were tested using the trained network.

For external validation, CT image sets from two institutions were used. Manual annotation and extraction were performed in the same way by radiologists with 6 and 12 years of experience in abdominal radiology. The 8 differently trained CNN models were applied to the external CT dataset without training. The final classification through a CNN algorithm was determined using the average of the network outputs from the 8 trained models as the image-level probability of acute appendicitis. The test procedures for the internal and external datasets are briefly summarized in Fig. 1 (lower). The CNNs were trained and tested using PyTorch on a system equipped with a single Nvidia GeForce GTX Titan RTX graphics processing unit^13^.

### Statistical analysis

With a 0.5 cut-off value of the image-level probability of acute appendicitis, true positives (TP), false positives (FP), true negatives (TN) and false negatives (FN) for the diagnosis of acute appendicitis in patients with acute abdominal pain were calculated for datasets from three institutions. The diagnostic performance of the algorithm for the CNN-based classification of acute appendicitis was evaluated on the basis of the sensitivity, specificity, and accuracy for each of the 8 test sets and for all subjects in the internal validation. For the external validation, the diagnostic performance of the algorithm for the CNN-based classification of acute appendicitis with the CT image sets from two institutions as the input was evaluated with regard to the sensitivity, specificity, and accuracy. To visualize the performance of the classification, an ROC curve analysis was performed. Statistical analysis was performed using Matlab 2018b (The Mathwork, Natick, MA, USA).

### Analysis of misjudgment

Misinterpreted features for FP and FN were analyzed through a review of the original CT images and a heatmap generated by gradient-weighted class activation mapping (Grad-CAM), which allows the features focused on by the trained CNN to be visualized^14^. The two radiologists that performed the manual annotation and extraction and a scientist that built the CNN-based algorithm participated in the misjudgment analysis.

## Results

**Figure 2:**
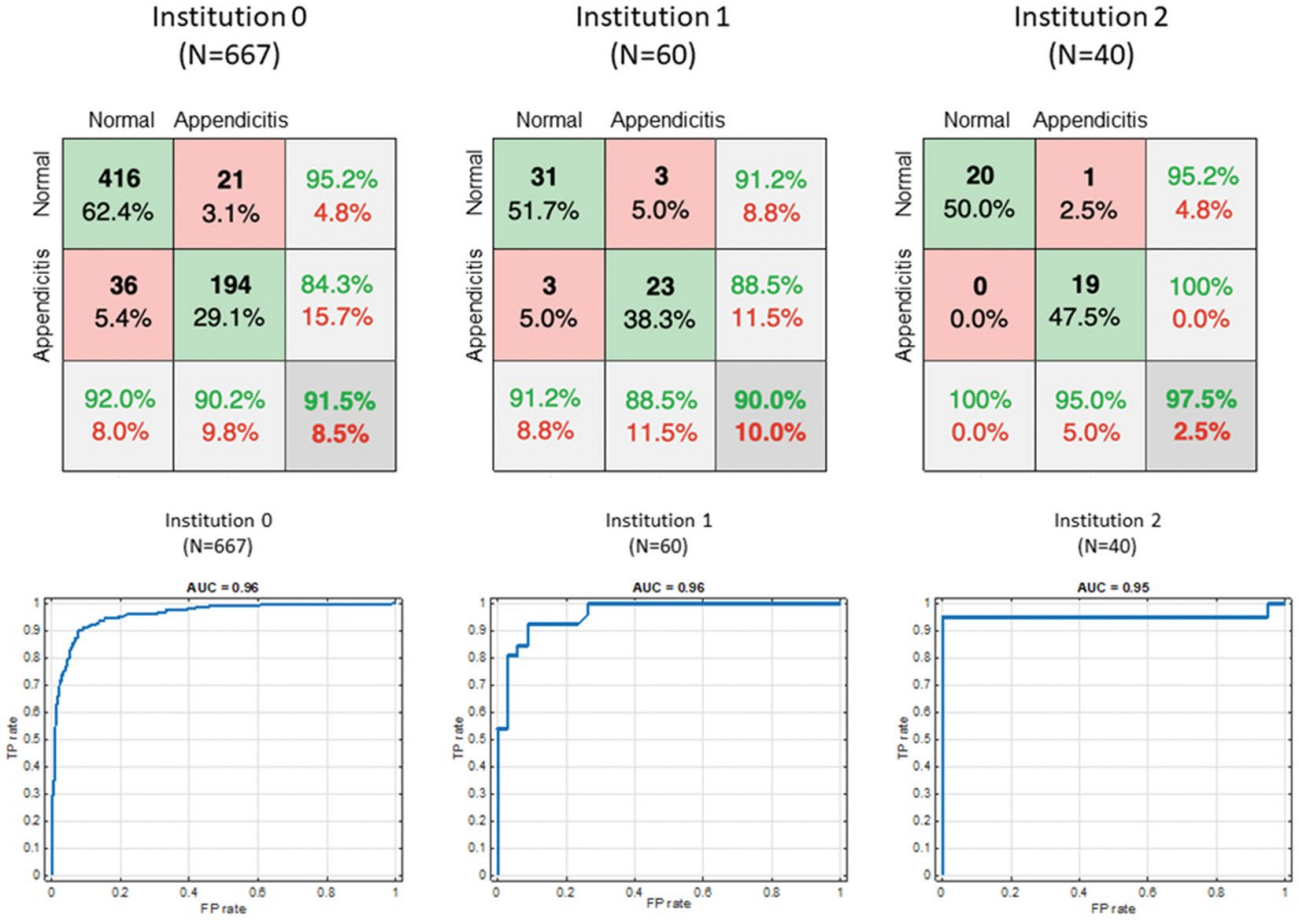
Confusion matrix (upper) used to diagnose acute appendicitis in the trained CNN using datasets for each of the three institutions and ROC curve analysis (lower) performed to diagnose acute appendicitis from a trained CNN using datasets from each of the three institutions considered (drawn by Microsoft PowerPoint and Matlab 2018b).

For training and internal validation, 667 CT image sets from 215 patients with acute appendicitis and 452 patients with a normal appendix were included (331 men and 336 women; mean age ± standard deviation (SD): 45.6 ± 22.2 years). The CT image set of the portal phase included 629 images, and the CT image set without contrast enhancement included 38 images. For external validation, 60 CT image sets of 26 patients with acute appendicitis and 34 patients with a normal appendix were included (25 men and 35 women; mean age ± SD: 45.9 ± 18.9 years) from institution 1. From institution 2, 40 CT image sets from 20 patients with acute appendicitis and 20 patients with a normal appendix were included (24 men and 16 women; mean age ± SD: 43.9 ± 20.8 years). The confusion matrix for the diagnosis of acute appendicitis in patients with acute abdominal pain using the trained CNN is shown in Fig. 2 (upper). The ranges of the outputs generated by a fully connected layer were (−6.311, 11.918) for output1 and (−11.887, 6.863) for output 2. With a 0.5 cut-off value of the image-level probability of acute appendicitis after application of the softmax function to the output value, the test results of internal validation using 8-fold validation for each of the 8 models and for all of them together are described in Table 1. The accuracy of the CNN-based classification algorithm of acute appendicitis was 91. 5% for all image sets (range, 86.9–94.7%). The diagnostic performance of the CNN-based algorithm for the diagnosis of acute appendicitis for all image sets was excellent: the sensitivity, and specificity were 90.2% (range, 85.2–96.3%), and 92.0% (range, 87.7–96.5%), respectively. Table 2 shows the results of the external validation. The diagnostic performance of the CNN-based algorithm for the diagnosis of acute appendicitis for the external CT dataset was also good to excellent. Figure 2 (lower) shows each ROC curve analysis conducted to diagnose acute appendicitis in patients with acute abdominal pain visiting the ER in each of the three institutions. The AUC was similarly high among the three institutions.

**Table 1:** Test results of the internal validation using 8-fold validation for each of the 8 training models.

Model (image set number)	TP	TN	FP	FN	Sensitivity	Specificity	Accuracy
1 (n = 84)	23	50	7	4	85.2	87.7	86.9
2 (n = 84)	26	54	3	1	96.3	94.7	94.7
3 (n = 84)	26	48	9	1	96.3	84.2	88.1
4 (n = 84)	24	55	2	3	88.9	96.5	94.1
5 (n = 84)	24	54	3	3	88.9	94.7	92.9
6 (n = 84)	23	55	2	4	85.2	96.5	92.9
7 (n = 84)	25	52	5	2	92.6	91.2	91.7
8 (n = 79)	23	48	5	3	88.5	90.6	89.9
Sum (n = 667)	194	416	36	21	90.2	92.0	91.5

TP, true positive; TN, true negative; FP, false positive; FN, false negative.

**Table 2:** Results of the external validation using CT data sets from two institutions for each of the 8 training models.

Model	Institution 1 (n = 60)			Institution 2 (n = 40)		
	Sensitivity	Specificity	Accuracy	Sensitivity	Specificity	Accuracy
1	88.5	91.2	90.0	90.0	90.0	90.0
2	88.5	91.2	90.0	95.0	80.0	87.5
3	92.3	88.2	90.0	95.0	100.0	97.5
4	80.8	91.2	86.7	95.0	95.0	95.0
5	88.5	94.1	92.0	95.0	95.0	95.0
6	88.5	88.2	88.3	90.0	95.0	92.5
7	88.5	88.2	88.3	90.0	90.0	90.0
8	88.5	91.2	90.0	90.0	90.0	90.0
Average	88.5	91.2	90.0	95.0	100.0	97.5

**Figure 3:**
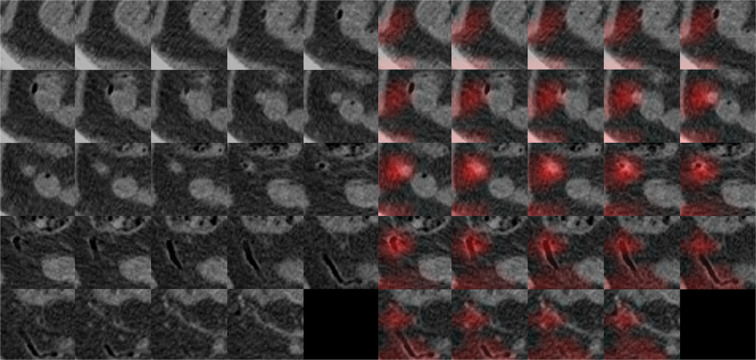
A 44-year-old woman who visited the ER with abdominal pain and a normal appendix. Original CT images within extracted 3D isotropic cubes show a normal appendix as an air-filled tubular structure (left side). Corresponding heatmap overlay using Grad-CAM highlights a normal appendix that is correctly recognized by the trained CNN (true negative) (right side).

**Figure 4:**
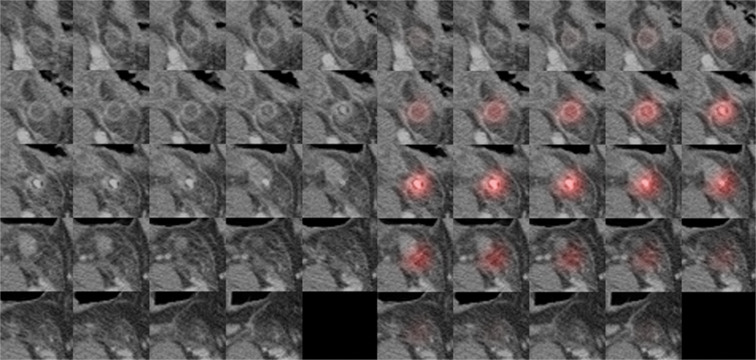
A 62-year-old woman who visited the ER with abdominal pain and was diagnosed with acute appendicitis. Original CT images within the extracted 3D isotropic cubes show a dilated appendix with wall thickening and appendicolith, compatible with acute appendicitis (left side). An inflamed appendix is highlighted on the heatmap overlay using Grad-CAM, which means the algorithm detected the appendix properly and diagnosed acute appendicitis accurately by assigning a given weight to the image location corresponding to the appendix (true positive) (right side).

**Figure 5:**
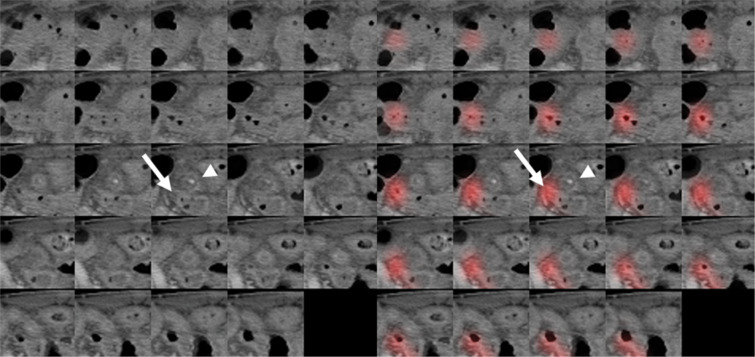
A 54-year-old woman who visited the ER with abdominal pain and was diagnosed with acute appendicitis. Original CT images within the extracted 3D isotropic cubes show an inflamed appendix with wall thickening and appendicolith, compatible with acute appendicitis (arrowhead) (left side). Corresponding heatmap overlay obtained using Grad-CAM showing that the terminal ileum (arrow) is incorrectly recognized by the trained CNN as a normal appendix (false negative) (right side).

**Figure 6:**
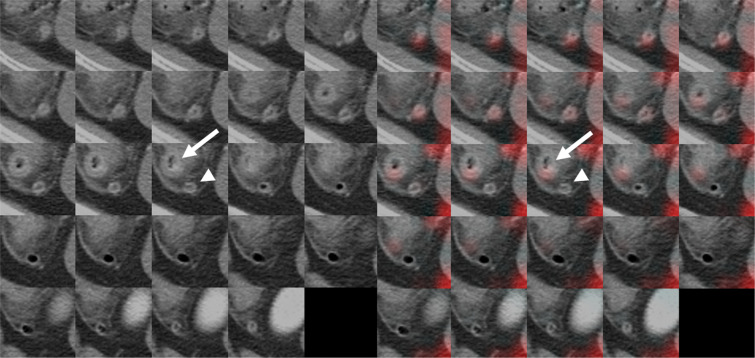
A 48-year-old man who visited the ER with abdominal pain and was diagnosed with cecal diverticulitis. Original CT images within the extracted 3D isotropic cubes show thick-walled diverticulum (arrow) in the cecum, fat stranding and peritoneal thickening for a normal appendix (arrowhead) (left side). Corresponding heatmap overlay obtained using Grad-CAM that represents an inflamed cecal diverticulum (arrow) that was recognized by the CNN as an inflamed appendix (false positive) (right side).

Figures 3 and 4 are heatmaps for each true negative and true positive case. According to an analysis of the misinterpreted features, the CNN-based algorithm mainly misjudged a collapsed ileum containing small air as a normal appendix in cases of FN (Table 3) (Fig. 5). For FP, the CNN incorrectly identified ileum with wall thickening or bowel dilatation as an inflamed appendix. Secondary changes caused by other inflammatory conditions except acute appendicitis, such as bowel wall thickening, severe fat stranding, fluid and peritoneal thickening, were features that were identified by the CNN-based algorithm and led to misinterpretation as FP (Fig. 6).

**Table 3:** Features emphasized by the CNN-based diagnosis algorithm causing misinterpretation of the data as either false positives or false negatives.

	Emphasized features		n
False Positives	Ileum	Small mesenteric fat	11
		Ileal wall thickening	3
		Ileal dilatation by fluid or air	8
		Normal	3
	Secondary changes by inflammation	Diverticulitis	8
		Duodenitis	1
		Acute cholecystitis	1
		Acute pyelonephritis	1
		Pelvic inflammatory disease	1
		Ischemic colitis	1
False Negatives	Ileum	Early acute appendicitis	15
		Appendiceal perforation with abscess	1
		Small mesenteric fat	4

## Discussion

The use of CT in the diagnostic workup of abdominal pain has become widespread^15,16^. CT is the primary and most appropriate imaging modality for evaluating patients with right lower quadrant pain and suspected appendicitis^17^. CT has shown high accuracy in detecting acute appendicitis and reduced negative appendectomies^18–20^. The high performance of CT can be achieved with an examination based on an appropriate CT protocol and accurate interpretation.

The appendix is a structure attached to the base of the cecum^3^. A normal appendix is not conspicuous and appears with a tubular, linear or curvilinear structure in radiologic imaging^21^. The position of the appendix is variable, with descending, pelvic, retroperitoneal, subcecal, preileal, postileal, and subhepatic locations^21^. Diverse conditions such as unusual appendix locations, scanty intraabdominal fat, prominent cecal wall thickening and pericecal fat stranding, small bowel dilatation, abscess formation adjacent to the right adnexa, and diseases that mimic appendicitis cause difficulty in detecting the appendix and diagnosing appendicitis^22^. Therefore, the detection and diagnosis of a normal appendix or an inflamed appendix are not easy for a clinician to carry out. However, radiologists are often not available during off hours, for example, at night in ERs. An alternative method that could carry out the roles of radiologists on their days off, introduce efficiencies to the risk prediction of acute appendicitis and provide decision support for clinical care of patients with abdominal pain in the ER would be very helpful. The deep learning method is used for this purpose.

The deep learning method is a class of machine learning algorithms using a representation-learning method with multiple levels of representation. Representation learning is a set of methods that allows a machine to be fed with raw data and to automatically discover the representations needed for detection or classification^23,24^. Therefore, deep learning allows the discovery of complicated structures in high-dimensional data with the requirement of very little engineering by human hands^24^. However, not so long ago, deep learning approaches were not extensively evaluated for the medical field, with challenges related to the sparse, noisy, heterogeneous, and time-dependent characteristics of medical data^25^. For the diagnosis of appendicitis, ANNs have been investigated in several studies^26–30^. In those studies, the diagnostic performance of the ANN was excellent in comparison with that of a clinical diagnosis, but only simple clinical data were used as inputs for the ANN.

With the rapid development of powerful parallel computing hardware, the availability of large quantities of labeled data and improved training techniques and architectures have enhanced large neural network training^23^. The ANN can handle vast amounts of radiologic imaging data. We applied artificial intelligence in the interpretation of CT data. As far as we know, radiologic imaging itself (CT) has not been used as the input for a CNN to diagnose acute appendicitis.

In this study, we evaluated the feasibility of a neural-network-based diagnosis algorithm of acute appendicitis using abdominopelvic CT for patients with acute abdominal pain visiting the ER as a specific circumstance with typical emergent conditions. We focused on the feasibility of early and accurate decisions regarding whether patients with acute abdominal pain had acute appendicitis or not without the intervening of human interpretation. Acute appendicitis could be differentiated from a normal appendix without expert radiologists for patients with acute abdominal pain visiting the ER.

False positive and false negative cases occurred in the diagnosis of appendicitis using a CNN-based diagnostic algorithm. Some cases of FN were abstruse because evidence of acute appendicitis was definite on the CT images and trained humans never misinterpreted these cases as normal (Fig. 6). Uncertainty exists regarding why the CNN-based algorithm misinterpreted the data as negative, but we cannot recognize which representations were adopted by the CNN directly. To determine misjudgment, we used Grad-CAM, which allowed visualization of the features focused on by the trained CNN, to be calculated in the last convolutional layers^14^. A heat map using a notable color was helpful in understanding the causes listed in Table 3.

The limitations of this study are as follows: We included a CT image set only for patients with a normal and an inflamed appendix. Patients who underwent surgical removal of the appendix and had a tumor in the appendix were excluded. Second, we trained and then tested the trained network using the 4 cm^3^ data, including the appendix region, manually extracted by radiologists. For practical applications, an automatic localization of the appendix region is necessary. Therefore, a future study is needed to develop an automatic localization algorithm of the appendix regions, along with a classification algorithm.

In conclusion, the CNN-based diagnosis algorithm may be feasible in diagnosing acute appendicitis using the CT data of patients visiting the ER with acute abdominal pain.
